# The Radical Cure of Reducible Hernia by Kocher's Method of Invagination, with Lateral Displacement of the Neck, of the Sac

**Published:** 1898-12

**Authors:** J. Lynn Thomas

**Affiliations:** Assistant-Surgeon to the Cardiff Infirmary, Consulting Surgeon to the Cardiff Provident Dispensary and to the Porth Hospital


### THE RADICAL CURE OF REDUCIBLE HERNIA BY KOCHER'S METHOD OF INVAGINATION, WITH LATERAL DISPLACEMENT OF THE NECK, OF THE SAC.

BY

J. LYNN THOMAS, F.R.C.S. Eng.,

*Assistant-Surgeon to the Cardiff Infirmary, Consulting Surgeon to the Cardiff Provident Dispensary and to the Porth Hospital.*

---

HAVING had the advantage of witnessing Prof. Kocher operate at his clinic for the radical cure of hernia—inguinal and femoral—by his own ingenious "displacement" method, I

think the operation, which is practised in this country at present, is in accordance with the description given in the English translation of his *Operative Surgery* (Stiles); but the latest modification seems to me to be of such importance, that a few remarks on the subject may prove of interest.

I have only had the opportunity of operating five times by this recent modification of the displacement method up to the present time; but the principle upon which it is founded seems so sound, and its execution so simple, that without doubt when it becomes better known it will come into general use.

The method as described in Kocher's *Operative Surgery* is fairly well known and is gaining in popularity; the surgeon who has practised that operation will at once appreciate the advantages of the modification: there was a potential peritoneal sheath or pouch in the displaced neck in the older method, but in the present method there is none.

I will assume that the operation is done as far as the complete separation of the hernial sac in the usual manner; the next step in the new method is to take hold of the free end of the hernial sac by means of the long curved forceps used in Kocher's operation and then invaginate the sac upon itself into the general peritoneal cavity, carrying the point of the forceps along the anterior abdominal wall until it is nearly opposite the anterior superior iliac spine of the side operated upon. The point of the forceps is now pushed against the abdominal wall and a small incision is made through the abdominal muscles down upon it, then the forceps carrying the hernial sac is pushed outwards, the sac is caught and pulled out through the wound after liberating the herniating forceps.

After making the sac quite taut, its neck is secured to the parietal peritoneum and transversalis fascia and the remainder is cut off, a suture closes the wound in the muscles and the crucial part of the operation is done. It takes less time to do it than to describe it.

The hernial sac, it will be observed, is turned outside in, and it is its peritoneal lining that is seen at the extrusion through the abdominal muscles. There is no ligaturing of the neck of the sac in the region of the internal abdominal ring, in fact there is neither a scar nor a depression left to invite the formation of another hernia in Hesselbach's triangle. The same condition is obtained by Macewen by his well-known method of folding the hernia sac upon itself like a fire-cracker; Kocher's method is much simpler to perform, and besides leaves nothing to give rise to a possible necrosis such as Macewen's pad has been known to do in some hands. Kocher's method can be adapted to any hernia with a separable sac, and probably it will in time be the only method for the radical cure of femoral hernia.

The essentially new point in its application to an inguinal hernia is the displacement of the neck of the hernial sac *internally* to the internal abdominal ring, instead of *externally* to its outer border (older method); the closure of the inguinal canal is the same as formerly, and the dressings of the wound are confined to a strip of gauze kept on by collodion.

Four months ago I operated upon a large double inguinal hernia for Dr. Treharne, by the above method. Sixteen days later the patient walked a quarter of a mile without any external hernia support, and at present the scar is as linear as if the patient had been in bed all the time or had worn a light (useless?) truss.

The "golden puncture" and the "royal stitch" have had their day in the operations for the radical cure of hernia; what we want to-day is a scientific "royal method," and I think the above method of Prof. Kocher is a step in the right direction for a large number of cases of reducible hernia.

The literature of the radical cure of hernia is already very extensive, and a great deal will be written upon the subject before surgeons are agreed as to the best methods of dealing with (*a*) the hernial sac, (*b*) the closure of the canal, (*c*) the nature of the ligature, and (*d*) the length of time a patient is to be kept in bed after operation. One surgeon pooh-poohs all elaborate methods of dealing with the sac, and is contented with a simple ligature round its neck, whilst his whole energy is spent in restoring the obliquity of the inguinal canal; another asserts that if the hernial sac be belaboured with skill and care it yieldeth cure, and the rest of the operation can be done by any seamstress.

One great authority states that the success of a radical cure depends upon allowing the wound to close by *second* intention, whilst another is perfectly certain that there is no chance of a cure except by *primary* union.

Lastly, A says the patient *must* be on his back for five or six weeks after the operation, and has to wear a light truss for some months afterwards; whilst B reports that his patients are always up on the eighth day after operation and never wear a truss or any other support. A and B, with the rest of authorities, are equally certain that "my method gives the best result possible," whilst that fortunate specimen of the species—the ordinary surgeon—gets excellent cures by somebody's method *modified by himself.* The reader will be relieved to know that I propose *not* to discuss fully and dispassionately the various views entertained by different schools, but will state that the patient who gets every factor in the production of hernia restored to a condition which does not invite further trouble is more fortunate than the one who gets only one of them put right.

I fail to see any objection to allowing the patients to get up and walk about after the skin sutures are removed, provided they have no cough and the case has run an aseptic course; we are not in the habit of seeing scar tissue in the abdominal wall show any trace of stretching for some months after any kind of operation performed, whatever method is employed in closing the wound, and whether it suppurates or not.

A thin narrow skin-scar can only be obtained by primary union, its permanency depends upon its being parallel with the normal lines of cleavage of Langer: our knowledge of the deeper scar tissue is not yet a very profound one.

The scrotum should never be incised in any case of hernia, as it cannot be rendered surgically clean: if the hernia constituents are very adherent to the contents of the scrotum, they, with the testicle, should be dislocated upwards into the groin-wound and separated upon a sterile towel, and the testis afterwards replaced after the fashion now adopted in the radical treatment of varicocele, spermatocele, and hydrocele.

One occasionally hears of a death from blood-poisoning after an operation undertaken for the radical cure of hernia: this indeed is a great calamity, and the surgeons who have had such a rude shock would have their confidence restored to a great extent by wearing sterilised thread gloves when operating. Since last February I have tried silk, taffeta, cotton, and thread gloves for different aseptic operations, and in hernia operations I find that I can operate with greater ease than with the bare hands, whilst feeling confident that the result will be primary union without even a suture-blush. The separation of the fascial layers off a hernial sac is more easily done, on account of the non-slipping-grip the glove gives in manipulation. I invariably use thread gloves now, because they are cheap, fit well, are easily washed, and do not get fluffy by repeated preparation for use. When dry they are easily put on, but if wet there is some trouble and delay.

---

